# Cheese starter cultures attenuate inflammation in the in vitro Caco-2 model

**DOI:** 10.3934/microbiol.2025017

**Published:** 2025-05-28

**Authors:** Deepa Kuttappan, Sulthana Humayoon Muttathukonam, Mary Anne Amalaradjou

**Affiliations:** Department of Animal Science, University of Connecticut, 17 Manter Road, Storrs, CT 06269, USA

**Keywords:** inflammation, inflammatory bowel disease, intestinal epithelial cells, Caco-2, cheese starter cultures, anti-inflammatory potential, *in vitro*

## Abstract

Chronic inflammation is identified to be an underlying pathophysiology in different conditions including inflammatory bowel disease (IBD). Since the aberrant interaction of the mucosal immune system with the dysbiotic flora has been reported to contribute to IBD development, probiotics have been studied for potential prophylaxis and treatment. In this regard, fermented dairy foods are a rich source of probiotics and bioactive compounds. However, limited studies have determined the impact of fermented dairy products in the context of chronic inflammation. In particular, a potential role for dairy starter cultures is not well studied. Hence, in this study we evaluated the anti-inflammatory effect of two cheese starter cultures (*Lactococcus lactis* subsp. *lactis* M58 and *Streptococcus thermophilus* TA 61) in comparison with commercial probiotic strains (*Bifidobacterium animalis* subsp. *lactis* BB-12, *Lactobacillus acidophilus* LA-5) using the Cmax-induced Caco-2 inflammation model. Specifically, we characterized their ability to attenuate inflammatory response via modulation of IL-8 secretion, NF-κB activation, barrier integrity (TEER), and tight junction gene expression. Overall, pre-exposure to the starter cultures before Cmax treatment significantly reduced the activation and nuclear translocation of NF-κB, compared to cytokine control (P < 0.05). Further, the reduction in pNF-κB was found to be associated with a significant reduction in IL 8 secretion (P < 0.05). Moreover, the cultures protected the Caco-2 monolayer from inflammation-induced increase in permeability by upregulating the genes associated with ZO-1 and occludin production. Furthermore, the protective effect of the starter cultures was comparable to that of the commercial probiotics with known anti-inflammatory properties. Therefore, cheese starter cultures could be a potential strategy against chronic gut inflammation.

## 1. Introduction

Inflammation is a physiological process directed at healing and maintenance of homeostasis [1],[2]. With multifactorial etiology, inflammation can be initiated by stimuli such as pathogens, chemical irritants, nutritional imbalance, and cell injury [2]. Although intended as a protective mechanism, when inflammation becomes chronic, it can cause severe and irreversible complications. In fact, chronic inflammation is identified to be an underlying pathophysiology in different conditions including inflammatory bowel disease (IBD) [3]. IBD is a progressive immune-mediated disease of the intestinal tract characterized by uncontrolled, relapsing inflammation leading to bowel damage [4]. Though the exact etiology of IBD is unclear, it is hypothesized that the disease originates from genetic susceptibility to dysregulated interaction between the immune system and the enteric commensal flora in the compromised gut. In fact, the two central features associated with IBD are a defective epithelial barrier and an exaggerated immune response [5]–[7].

Although different cell types contribute to inflammation in IBD, the intestinal epithelial cells are known to play a critical role in pathogenesis. The intestinal epithelial cells (IECs) have a strategic position at the interface between the luminal environment and the internal milieu [8]. IECs institute bidirectional interactions with the underlying immune cells and contribute to the mucosal inflammatory response [9]. Besides this, the IECs form an impermeable polarized monolayer along the gut wall in the absence of specific transporters. The intercellular space is furthermore sealed by junctional protein complexes, of which the tight junctions are located at the most apical pole of the epithelial cells. Tight junctions are the main gatekeepers of paracellular space and can mediate the permeability of ions and small molecules up to 20 kDa. The adherens junctions and desmosomes, in contrast, form strong adhesive bonds and are primarily responsible for maintaining tissue cohesion and integrity [10],[11]. The intact tight junctions between the epithelial cells are responsible for maintaining selective epithelial permeability in the intestine. When this is challenged as in IBD, pathogens, intestinal contents, and toxins can gain access into the epithelial layers, leading to sustained inflammation [12].

When inflamed, the mucosal immune cells are activated leading to IEC response and further barrier disruption [13]–[15]. The intestinal macrophages and dendritic cells sense pathogen-associated molecular patterns (PAMPs) and activate signal pathways, such as NF-kB, producing proinflammatory cytokines, chemokines, and anti-microbial peptides [16]. Increased production of inflammatory mediators including IFN-γ, IL-1β, TNF-α, IL-6, IL-8, IL-17A/F, IL-21, and IL-22 are observed in the intestine of IBD patients [17],[18]. Similarly, Caco2 cells exposed to a specific combination of inflammatory mediators, IL-1β, TNF-α, IFN-γ, and LPS, are found to mimic the gut inflammation [8].

Since the aberrant interaction of the mucosal immune system with the dysbiotic flora has been reported to contribute to IBD development, probiotics have been studied for potential prophylaxis and treatment [19]. Probiotics belonging to the lactic acid bacteria (LAB) group and in particular to the genera *Lactobacillus*, *Lactococcus*, and *Bifidobacterium* are reported to have therapeutic properties in IBD [19]–[21]. Besides supplementation of live probiotics, foods supplemented with the strains or fermented using these cultures are also shown to exert a protective effect against IBD [22],[23]. Among the different fermented foods, dairy products constitute a significant portion of our daily diet. These products are a rich source of probiotics, and prebiotic and bioactive compounds [24]. However, limited studies have determined the impact of fermented dairy products in the context of chronic inflammation including IBD [23]. Among the common bacteria associated with fermented dairy products, starter cultures including *Streptococcus thermophilus*, *Lactobacillus acidophilus*, and *Lactococcus lactis* are reported to be ingested in high concentrations in fermented milk and cheese [25]–[28]. Beyond their role in food fermentation, select starter culture strains have been shown to exert a gut protective effect [29]–[31]. Along these lines, in this study, we determined the anti-inflammatory potential of commercial cheese starter cultures (*Streptococcus thermophilus* TA-61 and *Lactococcus lactis* subsp *lactis* M-58) in comparison to established probiotic strains (*Bifidobacterium animalis* subsp. *lactis* BB-12 and *Lactobacillus acidophilus* LA-5) using an *in vitro* model simulating active inflammation associated with IBD.

## 2. Materials and methods

### 2.1. Culture condition for probiotics and starter cultures

Commercial probiotics, namely, *Bifidobacterium animalis* subsp. *lactis* BB-12 (BB) and *Lactobacillus acidophilus* LA-5 (LA), were kindly donated by Chr Hansen (Hoersholm, Denmark). The cheese starter cultures, *Streptococcus thermophilus* TA-61 (TA; Danisco A/S, Copenhagen, Denmark) and *Lactococcus lactis* subsp *lactis* M-58 (M; Danisco A/S, Copenhagen, Denmark) were obtained from Dairy Connection Inc. (Madison, WI, USA). All cultures except BB were grown in de Man, Rogosa, and Sharpe (MRS) broth (Difco, Sparks, MD, USA) under aerobic conditions at 37 °C for 24 h [32],[33]. *Bifidobacterium animalis subsp. lactis* (BB, Chr Hansen) was grown under anaerobic conditions at 37 °C in MRS containing 0.2 g/L lithium chloride and 0.3 g/L sodium propionate [34]. The bacterial count in these cultures was determined by plating 0.1 ml portions of appropriate dilutions on MRS agar (Difco, Sparks, MD, USA) with incubation at 37 °C for 24 h. The cultures were sedimented by centrifugation (3600 g, 15 min, 4 °C), and the pellets were washed twice with sterile PBS (pH 7.2). The pellets were resuspended in sterile Dulbecco's modified Eagle's medium supplemented with 20% fetal bovine serum to obtain the desired bacterial load (6 log CFU/mL).

### 2.2. Caco-2 cell culture

Caco-2 cells were obtained from ATCC (ATCC® HTB-37™) and were between passages 30–40 for all experiments (Manassas, VA, USA). Cells were cultured in Dulbecco's modified Eagle's medium supplemented with 20% fetal bovine serum. Cells were incubated at 37 °C and 7% CO_2_, and were subcultured at 80–90% confluence every 5–7 days. Once confluent, cells were detached with trypsin, counted, and seeded at a density of 1 × 10^5^ cells per mL wither on 12-well plates or on polycarbonate membrane Transwell inserts with 0.4 µm pore size (Corning, Inc; Lowell, MA, USA) for further assays. Seeded cells were cultured for 21 days to reach differentiation, and growth media was refreshed every 2–3 days [35],[36].

### 2.3. In vitro intestinal inflammation model

Caco-2 monolayer (Human intestinal epithelial colon carcinoma cell line, American Type Culture Collection) was cultured in Dulbecco's modified Eagle's medium supplemented with 20% fetal bovine serum in 12-well plates at 37 °C in the presence of 7% CO_2_ for 21 days. Following differentiation, Caco-2 cell monolayers (1 × 10^5^ cells/well) were exposed to the different treatments at (~6 log CFU/well; Table 1) for 24 h [37]. In addition, uninoculated monolayers were set up as controls. The monolayers were then washed and treated with the cytokine cocktail (Cmax; IL-1β-25 ng/mL, TNFα-50 ng/mL, IFNγ-50 ng/mL, and LPS-10 µg/mL; Thermo Fisher Scientific, Waltham, MA, USA; [8]) for 24 h to stimulate an inflammatory response.

**Table 1.** Experimental groups. Monolayers were exposed to the different cultures for 24 h @ 6 log CFU/well followed by treatment with Cmax (IL-1β-25 ng/mL, TNFα-50 ng/mL, IFNγ-50 ng/mL, and LPS-10 µg/mL) for 24 h. Samples were then processed for further analysis.

Group	Treatment
Control	Untreated (no culture, no Cmax)
Cmax	Monolayer treated with cytokine cocktail
BB	Monolayer exposed to *Bifidobacterium animalis* subsp. *lactis* BB-12 (BB) alone
LA	Monolayer exposed to *Lactobacillus acidophilus* LA-5 (LA) alone
M	Monolayer exposed to *Lactococcus lactis* subsp. *lactis* M-58 (M) alone
TA	Monolayer exposed to *Streptococcus thermophilus* TA-61 (TA) alone
BBCmax	Monolayer exposed to BB and treated with Cmax
LACmax	Monolayer exposed to LA and treated with Cmax
MCmax	Monolayer exposed to M and treated with Cmax
TACmax	Monolayer exposed to TA and treated with Cmax

### 2.4. IL-8 Assay

Caco-2 cells (1 × 10^5^ cells/mL) were grown in 12-well plates and treated as described above. Following stimulation with Cmax for 24 h, the cell culture supernatant was collected and stored at −80 °C until cytokines were analyzed. IL-8 estimation was performed using the IL-8/CXCL8 ELISA kit (R&D Systems Inc., Minneapolis, MN, USA; [38]).

### 2.5. Nuclear protein extraction and nuclear NF-kB p65 [pS536] assay

For this assay, Caco-2 cells (1 × 10^5^ cells/well) were cultured in 6-well plates at 37 °C in the presence of 7% CO_2_ for 21 days. The cells were then exposed to the different cultures for 14 h followed by stimulation with Cmax for an additional 24 h as described earlier (treatment scheme as in Table 1). Following this, nuclear proteins were isolated using NE-PER Nuclear and Cytoplasmic Extraction kit according to the manufacturer's protocol (Thermo Fisher Scientific, Waltham, MA, USA). Protein concentrations were determined using the Bradford protein assay (Bio-Rad). Fifty micrograms of nuclear protein from each sample were then subject to NF-kB p65 [pS536] ELISA as per the manufacturer's protocol (Thermo Fisher Scientific, Waltham, MA, USA; [39]–[41]).

### 2.6. Transepithelial electrical resistance (TEER) determination

Caco2 cells were seeded (1 × 10^5^ cells/well) on Transwell inserts in 12-well culture plates and allowed to differentiate as previously described [35],[36]. The monolayers were exposed to the cultures and stimulated with Cmax as described earlier (Table 1). TEER of Caco-2 cells before and after treatment was measured using a Millicell ERS system (Millipore, Billerica, MA, USA). An insert without cells was used as a blank and its mean resistance was subtracted from all samples. For untreated, fully differentiated monolayers, TEER values were routinely 300–500 Ωcm^2^.

### 2.7. Real time quantitative PCR (RT-qPCR) for tight junction gene expression

To understand the effect of probiotics and starter cultures on tight junction genes, RNA was isolated from the Caco-2 cells following exposure to the different experimental groups (Table 1), using the Qiagen RNeasy according to the manufacturer's instructions (Qiagen). cDNA was synthesized using the Bio-Rad iScript cDNA synthesis kit (Bio-Rad, Hercules, CA, USA). RT-qPCR analysis of the genes associated with tight junction protein expression (ZO-1, Occludin) was performed [35] and normalized against GAPDH gene expression. The relative fold change in gene expression was calculated using the 2−ΔΔCt method [42].

### 2.8. Statistical analysis

Each experiment was set up as a completely randomized design with three independent trials. All trials were performed in duplicate, and the results are presented as the mean ± standard error (SEM). The data were analyzed using the GraphPad Prism (v.10.1.1). One-way analysis of variance (ANOVA) was performed followed by Tukey-Kramer post hoc test for multiple comparisons amongst means. A *p* ≤ 0.05 was considered to be statistically significant.

## 3. Results and discussion

This study determined the potential anti-inflammatory properties of lactic cultures including starter cultures using an in vitro intestinal inflammation model. Since the IECs are reported to play a critical role in the pathophysiology of IBD, we employed the Caco-2 model. Further, the differentiated Caco-2 cell culture model is known to express tight junctions, cell surface receptors, transporters, and biotransformation enzymes making it the most commonly used in-vitro model of human enterocytes [43],[44]. To simulate the inflammatory environment in IBD, we used a cytokine cocktail (Cmax) consisting of TNFα, IFNγ, IL-1β, and LPS. These inflammatory mediators are reported to be associated with initiating, mediating, perpetuating, and controlling intestinal inflammation and tissue injury in IBD [45]–[48]. Specifically, these cytokines are known to play a role in barrier disruption, increased permeation of LPS and further precipitation of the inflammatory response [49],[50]. Moreover, the concerted effect of the cytokine cocktail serves to represent the endogenous (cytokines) and exogenous (LPS) inflammatory stimuli required for IBD development as seen in the acute phase of the condition [8].

### 3.1. Pre-exposure to starter cultures reduces Cmax-induced IL-8 secretion by differentiated Caco-2 cells

**Figure 1.**
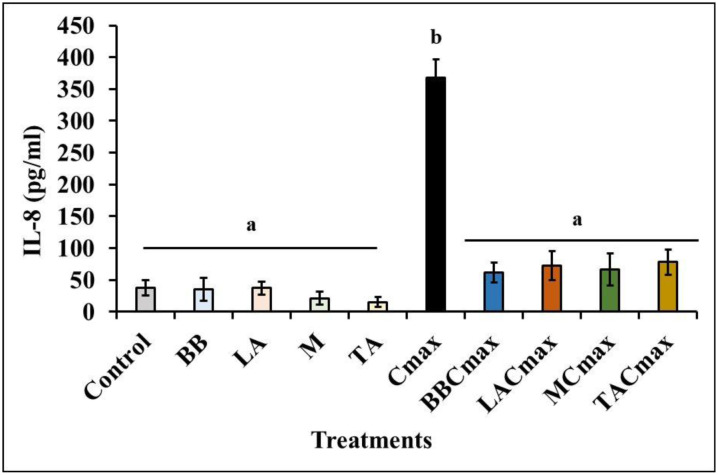
Pre-exposure to starter cultures (M, TA) and probiotics (BB, LA) decreases IL-8 production by differentiated Caco-2 cells exposed to inflammatory stimuli. Data is presented as means ± SD. Bars with different superscripts are significantly different from each other at *p* ≤ 0.05.

IL8 is a major chemokine active in IBD and involved in the chemotaxis of neutrophils and granulocytes to the inflammation site [51],[52]. Further, use of the cytokine cocktail (Cmax) has been shown to induce IL-8 secretion in a dose-dependent manner [8]. Further, literature demonstrates a key role for IL-8 in the pathophysiology of ulcerative colitis, a major type of IBD. Given this, we determined IL-8 levels in our inflammatory model following exposure to lactic cultures and administration of Cmax. As seen in Figure 1, in the Control samples (uninflamed, healthy cells, negative control), we observed a basal IL-8 concentration of 37.69 ± 12.20 pg/mL. Exposure of the Caco-2 cells to the different test strains (BBC, LCC, TAC, MC; Table 1) by themselves did not result in any significant change in IL-8 levels (15.4–37.3 pg/mL) when compared to the Control (37.69 pg/mL). On the other hand, as previously reported [8], administration of Cmax led to significant increase in IL-8 levels (p ≤ 0.0001). When compared to the basal level in the Control group, IL-8 levels were almost ten times higher (367.22 ± 29.82 pg/mL) in the Cmax group (positive control). On the other hand, pre-exposure to the test strains prior to Cmax administration significantly reduced IL-8 secretion in the Caco-2 monolayers (Figure 1). Specifically, IL-8 concentrations were 61.99 ± 15.62, 72.57 ± 22.69, 66.48 ± 25.13, and 78.28 ± 20.03 pg/mL in the BBCmax, LACmax, MCmax, and TACmax groups, respectively. Furthermore, the IL-8 levels in the treatment groups were not found to be significantly different from the control, indicating the potential anti-inflammatory effect of these strains. In addition, cheese starter cultures (TA, M) were equally as effective as the established probiotic strains (BB, LA) in protecting the monolayer from Cmax-mediated IL-8 production (Figure 1).

IL-8 is one of the most extensively studied chemokines produced by different cell types including IECs, neutrophils, T lymphocytes, and macrophages [53]–[56]. More particularly, although barely detectable in unstimulated cells, IL-8 levels can increase by 10–100 times following inflammation such as activation by TNF-α, IL-6, IFN-γ, and LPS [57],[58]. This is in line with our results which demonstrate a ~100-fold increase in IL-8 levels in the in-vitro IEC model following administration of the cytokine cocktail (Figure 1). IL-8 is known to exert a pleiotropic role in inflammatory response via the recruitment and activation of neutrophils and granulocytes to the site of inflammation resulting in intense and chronic immune response and tissue damage [59]. Thus, it plays a key role in chronic inflammatory conditions including IBD and in particular ulcerative colitis [58],[60],[61]. Moreover, increased IL-8 secretion is well documented in the colonic mucosa of patients with active inflammation with a direct correlation to severity of colitis [62],[63].

Given its critical role in inflammation, several studies have reported a protective effect following attenuation of proinflammatory cytokine production and downstream signaling [64]–[66]. Related to our results, application of probiotics has been proven to be beneficial in IBD when used alone or in combination with conventional drugs [67]–[69]. Specifically, regular consumption of kefir containing lactobacilli was seen to reduce inflammation and improve quality of life in patients with IBD [70]. Moreover, probiotic supplements containing *Lactobacillus* and *Bifidobacterium* strains were shown to be more effective in inducing remission in IBD [68]. For instance, administration of BB and LA was shown to suppress IL-8 secretion by TNF-α-stimulated HT-29 cells in vitro while improving colitis in a DNBS-induced mouse model [71]. Similarly, this anti-inflammatory effect was also observed following *Salmonella* infection in a gnotobiotic piglet model [72]. Our results align with these findings as seen from the significant reduction in IL-8 production in the inflamed IECs in vitro. More importantly, we observed that the starter cultures (TA and M) exerted a significant anti-inflammatory effect on the Caco-2 cells similar to the known probiotic strains (BB, LA; Figure 1). This is significant since previous studies using live cultures of related LAB strains derived from dairy products were shown to reduce Il-8 production in vitro and attenuate inflammation in vivo [73]–[75]. This highlights a potential role for dairy starter cultures in mediating the gut-health-promoting role of fermented dairy foods [23].

### 3.2. Pre-exposure to starter cultures reduces Cmax-induced NF-kB activation in differentiated Caco-2 cells

NF-κB is activated by viral and bacterial infections, necrotic cell products, DNA damage, oxidative stress, and pro-inflammatory cytokines [76],[77]. When the stimulation occurs, the activated p65 subunit of NF-κB translocates to the nucleus and binds to the response elements transactivating the expression of pro-inflammatory cytokines including IL-8 [78]. In effect, NF-κB binding to the IL-8 promoter element is required for its transcriptional activation. Therefore, any stimuli that modulates NF-κB activity can also modify IL-8 induction. Specifically, inhibition of NF-κB activation can reduce transcriptional activation of IL-8 thereby attenuating the inflammatory response [53]. Thus, given our previous observation that exposure to starter cultures and probiotics significantly reduced IL-8 production in stimulated Caco-2 cells, as a next step we determined nuclear pNF-kB levels using ELISA.

**Figure 2.**
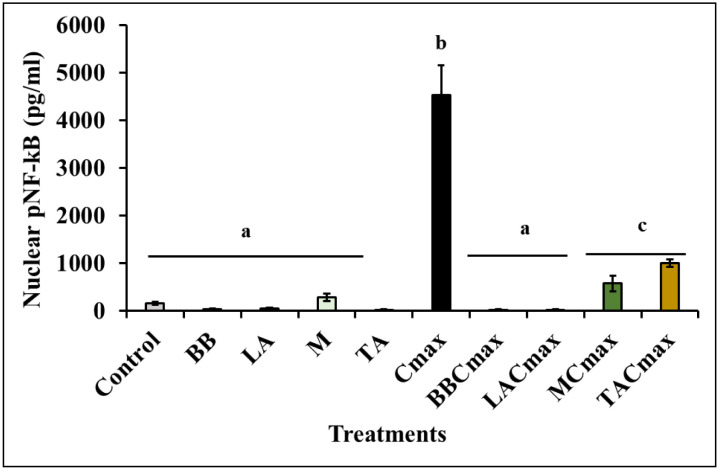
Pre-exposure to starter cultures (M, TA) and probiotics (BB, LA) attenuates NF-kB activation in differentiated Caco-2 cells exposed to inflammatory stimuli. Data is presented as means ± SD. Bars with different superscripts are significantly different from each other at *p* ≤ 0.05.

As seen with the IL-8 assay, exposure of the Caco-2 monolayers to the lactic cultures by themselves did not induce any significant activation of NF-kB when compared to the control (*p* > 0.05; Figure 2). Whereas treatment with Cmax led to a significant activation of NF-kB as evident from the increased pNF-kB levels in the nuclear fraction (4523 ± 628.6 pg/mL) when compared to the control (154.6 ± 31.03 pg/mL; *p* < 0.0001; Figure 2). However, pretreating the Caco-2 cells with probiotics and cheese cultures followed by Cmax stimulation led to significant attenuation of NF-kB activation as seen from the reduced pNF-kB levels in the nuclear fraction of the cell lysate in comparison to Cmax alone (*p* < 0.0001; Figure 2). For instance, pNF-kB levels in the inflamed IECs exposed to the cheese starter cultures (MCmax, TACmax) ranged from 572–994 pg/mL as opposed to 4523 pg/mL in the Cmax group (Figure 2). These results demonstrate that starter cultures potentially exert their anti-inflammatory effect by inhibiting NF-kB-mediated signaling and subsequent cytokine production including IL-8. Further, their anti-inflammatory effect was comparable to that of the established probiotics, namely BB and LA (Figure 2).

NF-kB, as a regulator of gene transcription, is involved in the imbalance of activation of pro-inflammatory and anti-inflammatory signaling pathways in the gut [79]. In line with our findings, Heuvelin et al. [80] demonstrated that *Bifidobacterium breve*-conditioned medium inhibited IL-8 secretion by HT29-19A epithelial cells through the NF-kB pathway. Further, consumption of yogurt fermented with starter culture (YF-L702) containing *Streptococcus thermophiles* and *Lactobacillus delbrueckii* subsp. *bulgaricus* and co-inoculated with BB-12 was shown to reduce pro-inflammatory cytokines in cultured peripheral blood monocytes from healthy individuals following in-vitro LPS stimulation [81]. Similar reduction in inflammatory mediators in LPS-stimulated RAW264.7 macrophages by *Bifidobacterium adolescentis* was associated with reduced phosphorylation of I-κBα subunit of NF-kB [82]. Likewise, *Lactobacillus casei* and *Bifidobacterium lactis* NCC362 were seen to inhibit p65 nuclear translocation through a decrease in I-kBα ubiquitination and degradation, thereby attenuating NF-kB-mediated inflammatory signaling in HT-29 cells [83],[84]. Overall, the inhibition of NF-κB activity may explain the reduction in IL-8 production from stimulated IECs pre-exposed to probiotics and starter cultures in our study.

### 3.3. Pre-exposure to starter cultures protects the Caco-2 monolayer from Cmax-induced loss in epithelial barrier integrity

**Figure 3.**
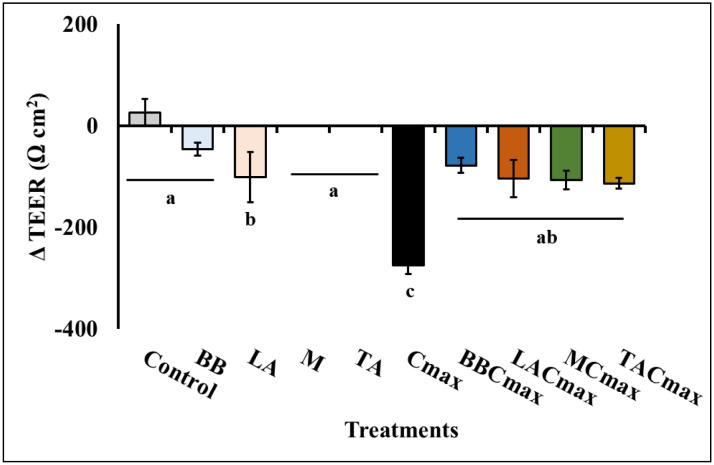
Pre-exposure to starter cultures (M, TA) and probiotics (BB, LA) mitigates barrier permeability in differentiated Caco-2 monolayers exposed to inflammatory stimuli. Data is presented as means ± SD. Bars with different superscripts are significantly different from each other at *p* ≤ 0.05.

A compromised intestinal epithelium is a feature observed in different intestinal inflammatory conditions including IBD and celiac disease [85]. Studies have revealed several defects in the specialized components of the mucosal barrier, from the mucus layer composition to the adhesion molecules that regulate paracellular permeability in IBD patients [86]. Given the critical role for barrier integrity in chronic inflammation, several studies have demonstrated the efficacy of different probiotics including *Escherichia coli* Nissle 1917, *Bifidobacterium*, *Lactobacillus rhamnosus* GG, and the multispecies VSL#3 in preventing leaky gut in IBD [86]–[88]. Along these lines, we evaluated the effect of our treatments on differentiated Caco-2 cell barrier integrity using TEER measurements. Except for LA, exposure to the starter cultures and/or the probiotics alone did not result in any significant reduction in TEER in the Caco-2 monolayers (*p* > 0.05). However, stimulation with the cytokine cocktail (Cmax) led to a 62% reduction (-275.33 ± 16.21 Ωcm^2^, Figure 3) in TEER from the initial value prior to treatment application. Van De Walle et al. [8] reported a similar reduction in TEER and loss in Caco-2 barrier integrity following treatment with Cmax.

On the other hand, pre-exposure to the starter cultures and probiotics protected the monolayer from Cmax-induced increase in permeability and reduction in TEER (*p* < 0.001, Figure 3). For example, in the TACmax and MCmax groups, we only observed a 25% reduction in TEER as opposed to the 62% reduction observed in the Cmax group. These data indicate that in addition to attenuating inflammation, cheese starter cultures can also protect the IECs from inflammation induced loss in barrier integrity. Further, their effect was comparable to that of the commercial probiotic strains (BB and LA) with proven anti-inflammatory and gut protective effects [71],[89]. A similar protective effect was reported following exposure of stimulated Caco-2 cells to lyophilized yogurt [35]. Also, it has been suggested that the protective effect of the dairy culture *S. thermophilus* NCIMB 41,856 is mediated by its ability to maintain the mucosal barrier thereby allowing healing of colitis [90]. Similar observations are also reported for other dairy-derived cultures including *Lactobacillus helveticus*, *Lactobacillus delbrueckii*, and *Lactococcus lactis* in in-vitro and in-vivo models [23].

### 3.4. Pre-exposure to starter cultures positively promotes Caco-2 monolayer barrier integrity by modulating tight junction gene expression

The intestinal epithelial barrier is comprised of a series of intercellular junctions made up of tight junctions, adherens junctions, and desmosomes [85]. Of these, the tight junctions primarily regulate paracellular permeability through a network of proteins including tight junction-associated marvel proteins such as occludin and intracellular scaffold proteins namely zonula occludens (ZO; [91]–[93]). Occludin is a transmembrane protein that is critical for localization of tight junctions [94]. Further, the carboxy terminal end of occludin contains the binding site for ZO-1. The ZO group of proteins (ZO-1, ZO-2, ZO-3) interacts with actin and help link the tight junction strands with the cytoskeleton [95]. This association of the cytoskeleton is critical for the maintenance of tight junction function and regulation of paracellular permeability. Toward this, abnormal tight junction structure and a down-regulation and redistribution of proteins including ZO-1 and occludin have been associated with loss in barrier permeability seen in conditions such as IBD [96]–[98]. Given the critical role of the tight junctions in barrier integrity and the results of our TEER assays, we performed gene expression assays to elucidate the effect of probiotics and starter cultures on the expression of tight junction protein-coding genes, namely *OCLN* and *TJP-1* (ZO-1).

**Table 2.** Pre-exposure to starter cultures (M, TA) and probiotics (BB, LA) promotes epithelial barrier integrity by modulating tight junction protein gene expression. Data is presented as means ± SD. For each gene, different superscripts indicate a significant difference between treatments at *p* ≤ 0.05.

Treatments	Relative fold change in gene expression	
	*TJP1*	*OCLN*
Control	1.07 ± 0.03^a^	1.12 ± 0.05^a^
BB	1.75 ± 0.14^b^	2.03 ± 0.24^b^
LA	1.85 ± 0.22^b^	1.75 ± 0.35^b^
M	2.11 ± 0.13^b^	1.80 ± 0.29^b^
TA	2.00 ± 0.15^b^	1.63 ± 0.09^a^
Cmax	-1.82 ± 0.21^c^	-2.16 ± 0.20^c^
BBCmax	1.54 ± 0.21^ab^	1.66 ± 0.25^ab^
LACmax	1.75 ± 0.17^b^	1.64 ± 0.39^ab^
MCmax	1.80 ± 0.17^b^	1.36 ± 0.23^a^
TACmax	1.66 ± 0.15^b^	1.34 ± 0.04^a^

As seen from Table 2, treatment with Cmax significantly reduced *TJP1* and *OCLN* expression by −1.82 ± 0.21 and −2.16 ± 0.19 fold, respectively, when compared to the Control (*p* ≤ 0.05). However, pre-exposure to the starter cultures and commercial probiotics helped protect the monolayer from Cmax induced downregulation in *TJP-1* and *OCLN* expression (*p* ≤ 0.05). Specifically, we observed that pre-exposure to the starter cultures and probiotics significantly increased *TJP-1* and *OCLN* expression in the healthy monolayer when compared to the Control (*p* ≤ 0.05; Table 2). Further, once these monolayers were stimulated using Cmax, the target strains continued to protect the monolayer from the inflammation-mediated attenuation of tight junction gene expression. Pre-exposure to the cheese starter cultures prior to Cmax treatment helped maintain *TJP-1* and *OCLN* levels like that of the unstimulated cells exposed to TA or M. In addition, as seen with our previous assays, the improvement in tight junction gene expression was comparable to that of the commercial probiotic strains BB and LA. Moreover, these results could help explain the improvement in TEER measurements seen with the starter culture and probiotic-treated groups when compared to Cmax alone (Figure 3).

Our findings are in agreement with increased mRNA expression of Caco-2 tight junction proteins and improved intestinal barrier function after exposure to *Lactobacillus plantarum* MB452 [99]. Similarly, treatment with lyophilized yogurt containing *Lactobacillus bulgaricus* and *Streptococcus thermophilus* was seen to increase claudin-1, ZO-1, and occludin mRNA levels in Cmax-stimulated Caco-2 cells [35]. Likewise, *Bifidobacterium dentium* N8 was shown to alleviate LPS-induced intestinal barrier injury in Caco-2 monolayers by regulating tight junction gene expression [100]. Besides these in-vitro studies, supplementation of dairy derived cultures including *S. thermophiles* MN-BM-A01 and *Propionibacterium freudenreichii* CIRM-BIA129 were seen to improve gut barrier integrity in the DSS-induced mice colitis model [90],[101]. Similarly, use of an *E. coli* Nissile strain engineered to carry zinc and indole-3-carbinol (ZI@EcN) on its surface was shown to significantly reduce inflammation in Caco-2 cells by restoring tight junction protein expression and restoring the epithelial barrier integrity [102]. In summary, none of our tested strains exerted any intrinsic pro-inflammatory effect on the Caco-2 monolayer. However, following the inflammatory stimulus, all tested strains exerted a significant protective effect on the IECs (*p* ≤ 0.05).

## 4. Conclusions

Overall, our data indicate that cheese starter cultures (*Streptococcus thermophilus* TA-61 and *Lactococcus lactis* subsp. *lactis* M-58) exert a significant protective effect against cytokine-mediated inflammation on IECs in vitro. Specifically, by attenuating NF-κB-mediated inflammatory signaling and chemokine production (IL-8), and upregulating tight junction gene expression, the starter cultures (TA and M) protected the Caco-2 monolayer from inflammation and loss in barrier permeability. Moreover, we also observed that the anti-inflammatory effect of the starter cultures was comparable to that of commercial probiotics (*Bifidobacterium animalis* subsp. *lactis* BB-12 (BB) and *Lactobacillus acidophilus* LA-5) with demonstrated anti-inflammatory effects in vitro and in vivo. Therefore, the cheese starter cultures, *Streptococcus thermophilus* TA-61 and *Lactococcus lactis* subsp. *lactis* M-58, could be employed as an adjunct therapy for inflammation associated with IBD in humans. However, further validation of their anti-inflammatory and gut-protective effects in vivo is warranted.

## Use of AI tools declaration

The authors declare they have not used Artificial Intelligence (AI) tools in the creation of this article.
